# Postindustrial Jute Waste as a Support for Nano-Carbon Nitride Photocatalyst: Influence of Chemical Pretreatment

**DOI:** 10.3390/polym16141989

**Published:** 2024-07-11

**Authors:** Milica V. Carević, Tatjana D. Vulić, Zoran V. Šaponjić, Nadica D. Abazović, Mirjana I. Čomor

**Affiliations:** 1 Vinča Institute of Nuclear Sciences, National Institute of the Republic of Serbia, University of Belgrade, Mike Petrovića Alasa 12–14, 11351 Belgrade, Serbia; tanja030@vin.bg.ac.rs (T.D.V.); mirjanac@vin.bg.ac.rs (M.I.Č.); 2 Institute of General and Physical Chemistry, Studentski Trg 12/V, 11158 Belgrade, Serbia; zsaponjic@iofh.bg.ac.rs

**Keywords:** carbon nitride, textile dye, photocatalysis, jute, post-industrial waste

## Abstract

Non-woven jute (NWJ) produced from carpet industry waste was oxidized by H_2_O_2_ or alkali-treated by NaOH and compared with water-washed samples. Changes in the structure of the NWJ, tracked by X-ray diffraction (XRD), showed that both chemical treatments disrupt hydrogen bond networks between cellulose Iβ chains of the NWJ fibers. Thereafter, nano-carbon nitride (nCN) was impregnated, using a layer-by-layer technique, onto water-washed jute samples (nCN-Jw), NaOH-treated samples (nCN-Ja) and-H_2_O_2_ treated samples (nCN-Jo). Analysis of the Fourier transform infrared spectroscopy (FTIR) spectra of the impregnated samples revealed that nCN anchors to the water-washed NWJ surface through hemicellulose and secondary hydroxyl groups of the cellulose. In the case of chemically treated samples, nCN is preferentially bonded to the hydroxymethyl groups of cellulose. The stability and reusability of prepared nCN-jute (nCN-J) samples were assessed by tracking the photocatalytic degradation of Acid Orange 7 (AO7) dye under simulated solar light irradiation. Results from up to ten consecutive photocatalytic cycles demonstrated varying degrees of effectiveness across different samples. nCN-Jo and nCN-Ja samples exhibited declining effectiveness over cycles, attributed to bond instability between nCN and jute. In contrast, the nCN-Jw sample consistently maintained high degradation rates over ten cycles, with a dye removal percentage constantly above 90%.

## 1. Introduction

In three Asian countries (India, Bangladesh and China), home to roughly 40% of the world’s population, 3.4 million metric tons [1] of jute are produced annually. Jute is conventionally employed in industries where mechanical stability is required since its fibers are robust, firm and non-stretchy. Approximately 80% of produced jute fibers are traditionally utilized as packaging material, while more than 15% are used as carpet backing and yarn [2].

Jute fibers have a complex chemical composition, with three main components: cellulose (up to 63%), hemicellulose (21–24%) and lignin (up to 12%), while the remaining part is divided between inorganic water solubles, waxes and fats [2]. Due to the similar carbohydrate origin and abundance of the hydroxyl groups, cellulose fibers (linked glucose units) and amorphous hemicellulose (a combination of C5 and C6 monosaccharide units) are easily coupled by hydrogen bonds [2]. Lignin, a complex polymeric structure built from guaiacyl, syringyl and 4-hydroxylphenyl propane units [3], making it rich in carbon–carbon bonds, is resistant to fragmentation and degradation [4,5,6] and is responsible for thermal resistance and rigidity of the jute cell fibers [6]. Usage of jute is somewhat hindered because of these characteristics, i.e., it cannot be used, for example, for apparel production. There are numerous examples in the literature of how its chemical, and consequently mechanical, properties can be altered by alkali or oxidative treatment, commonly used in the textile industry. Specifically, the removal of hemicellulose by alkali treatment results in a less dense and hard interfibrillar area, which permits fibrils to reorganize along the direction of tensile strain [7] and increases relative cellulose share and flexibility of the fiber. Furthermore, alkali-treated fibers have a greater capacity for sorption of moisture, a characteristic attributed to the increased availability of hydroxyl groups on cellulose fibers following hemicellulose removal [6]. Oxidative treatment, in addition to improving the whiteness of the fibers by removing their natural color, can also have two chemical effects [8]: oxidation of cellulose, which results in the cleavage of carbon–carbon bonds and the production of dialdehydes [9], and oxidative depolymerization of lignin through cleavage of C–C links [8,9,10]. Due to the abundance of the hydroxyl groups, jute fiber provides sufficient connection sites for oxide- and nitride-based semiconductor photocatalysts through hydrogen bonding, and as such can be very attractive for the application in photocatalysis as a carrier of photocatalysts, bypassing the common problem in heterogeneous photocatalysis, namely the retrieval of photocatalysts from the reaction mixture and their reuse in the consecutive processes.

Jute-based carpet waste, produced when a surplus of the weft yarns is trimmed off to provide equal carpet edges [11], can be transformed to non-woven material by the needle punch process. In our previous study [12], chemically untreated non-woven jute (NWJ) was utilized as a carrier of carbon nitride nanosheets (n-C_3_N_4_) and probed in photocatalytic degradation of three textile dyes. Fairly constant effectiveness in the three consecutive photocatalytic cycles proved the relatively strong and stable bonding between nano-carbon nitride (nCN) and untreated jute-based textile waste.

To compare the effects of various chemical treatments on the semiconductor bonding on the surface of the fiber and, in turn, on the photocatalytic effectiveness of the fixed photocatalyst, in this study, we treated NWJ produced from carpet industry waste through either an oxidative or an alkali treatment before applying nCN.

## 2. Materials and Methods

### 2.1. Materials

The following commercial chemicals were used: urea (VWR, Leuven, Belgium), acid orange 7 (AO7, Color index–15510, Cassella, Frankfurt, Germany), sodium hydroxide (Sigma Aldrich, Stenheim, Germany), hydrogen peroxide (Chemsolute, The Geyer, Renningen, Germany), acetic acid (Sigma Aldrich, Buchs, Switzerland). All chemicals were used as received. Non-woven jute was supplied by the company “Meteks” (Mladenovac, Serbia). In all experiments, ultrapure water (resistivity 18 MΩ cm) from Milli Q Water Systems was used.

### 2.2. Characterization

X-ray diffraction (XRD) patterns of all samples were collected using a Philips PW 1050 powder diffractometer (Philips, Amsterdam, Netherlands) (scanning technique: step size = 0.05, counting time = 5 s/step) with Ni-filtered Cu Kα radiation (λ = 0.1542 nm). The average crystallite size D (nm) was determined from XRD patterns according to the Scherrer equation (Equation (1)) [13]:

(1) $$D=\frac{k\lambda }{\beta \cos\theta }$$

where k is a constant equal to 0.94, λ is the wavelength of the X-rays equal to 0.1542 nm, ꞵ is the full width at half-maximum of the X-ray diffraction peak at θ, the Bragg angle of the peak. The Z-value, which indicates whether cellulose is Iα (Z > 0) or Iβ (Z < 0), was calculated from Equation (2) [13]:

(2) Z=1693d1−902d2−549

where d1 is the d-spacing of the Iβ (1-10) lattice plane and d2 is the d-spacing of the Iβ (110) lattice plane. The crystalline index (Cr.I.) was calculated using the empirical method proposed by the Segal equation (Equation (3)) [13]:

(3) $$Cr.I.=\frac{\left(I_{200}-I_{am}\right)}{I_{200}}\times 100$$

where I_200_ is the maximum intensity of the (200) diffraction and I_am_ is the intensity of the diffraction at 2θ ~ 18°.

Surface morphologies and qualitative chemical compositions over the jute sample surfaces were analyzed using a Scios 2 Dual Beam scanning electron microscope (SEM, Thermo Fisher Scientific, Waltham, MA, USA) equipped with an energy-dispersive X-ray spectroscope (EDS) at 10 kV of acceleration voltage. The samples were sputter-coated with gold (Au) and placed on a sample holder using double-sided adhesive carbon tape. The UV–Vis absorbance spectra were recorded in the spectral range from 200 to 900 nm using a UV-2600i spectrophotometer (Shimadzu Corporation, Kyoto, Japan).

Fourier-transform infrared (FTIR) spectroscopy measurements were performed by using a Nicolet 380 FTIR spectrometer (Thermo Fisher Scientific) in the attenuated total reflection (ATR) mode (Smart Orbit™ ATR attachment). FTIR spectra were taken in the spectral range from 4000 to 525 cm^−1^.

### 2.3. Sample Preparation

nCN: Nano-carbon nitride (nCN) samples were synthesized by thermal exfoliation of urea-based graphitic carbon nitride at 500 °C for 2 h, with a ramp rate of 5 °C/min [12]. The detailed procedure is given in the Supplementary Materials.

nCN-J: NWJ was utilized as the photocatalyst carrier. It was manufactured through a needle-punching process from carpet fringes comprised primarily of jute, with a small polyamide content, which were obtained as post-industrial waste from the carpet industry. The NWJ was cut into pieces measuring approximately 3 cm × 3 cm. Prior to the photocatalyst immobilization, the jute samples underwent various pretreatments:

Ja—The jute sample was treated with 30 mL of 1% NaOH (0.25 M) aqueous solution for 30 min at room temperature. Afterward, the sample was neutralized with 1% acetic acid and subsequently rinsed in distilled water (3 × 600 mL).

Jo—The jute sample was treated with 30 mL of 2% H_2_O_2_ aqueous solution for 30 min at room temperature and was then rinsed in distilled water (3 × 600 mL).

Jw—The jute sample was rinsed in distilled water (3 × 600 mL).

The jute samples’ masses were measured before and after drying at 80 °C for 20 h, as well as before and after the treatments with NaOH, H_2_O_2_ and H_2_O. The moisture contents and the weight losses which occurred as a result of the jute samples’ pretreatments were calculated using the direct gravimetric method. Results are shown in Table 1.

nCN-Ja, nCN-Jo and nCN-Jw: The nCN photocatalyst was immobilized onto the pretreated jute samples (Ja, Jo and Jw) as follows: 50 mg of nCN were dispersed in 25 mL of H_2_O using ultrasonication for 30 min. The photocatalyst dispersion was drop-coated layer-by-layer on the jute sample in a multistep process. In each step, 3 mL of the photocatalyst dispersion was added drop-by-drop onto the surface of the jute samples, followed by drying at 80 °C before the next step. Finally, the jute samples were rinsed several times with deionized water and dried at 80 °C.

### 2.4. Photocatalytic Activity Test

The photocatalytic performances of the nCN-J samples were investigated by monitoring the photodegradation of the textile dye AO7 (textile, acid, azo-dye; structural formula given in the Supplementary Materials) under simulated solar light. All details regarding performed photocatalytic tests are given in the Supplementary Materials. To determine the stability and reusability, the photocatalytic experiments were conducted in up to ten consecutive cycles. After each cycle, nCN-J samples were air dried at 80 °C and reused in the next cycle.

## 3. Results and Discussion

### 3.1. Characterization of the NWJ

**Figure 1:**
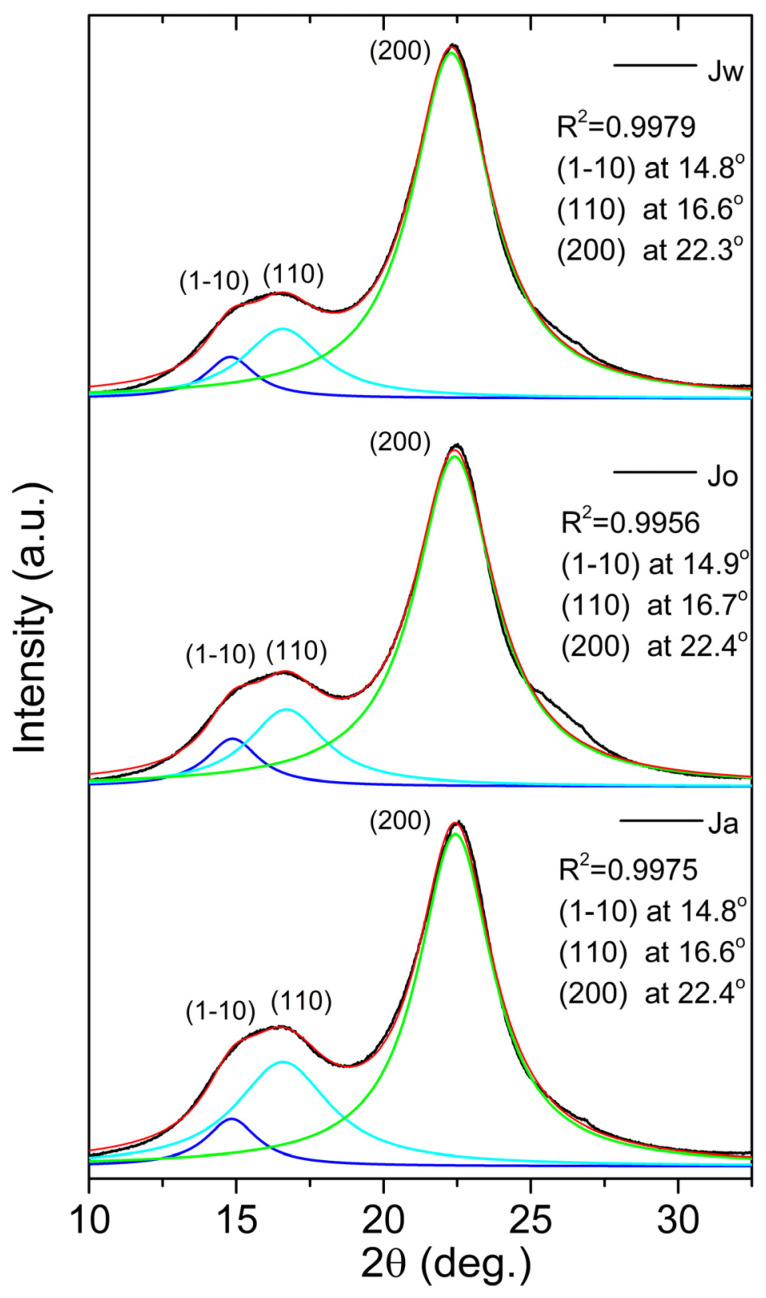
Deconvoluted XRD patterns of Jw (water-washed), Jo (treated with hydrogen peroxide) and Ja (alkali-treated) jute samples. Black line represents experimental data, red line is the best fit, while green, turquoise and blue line are fit peaks of (200), (110) and (1-10) diffractions, respectively.

As already noted, the three main components of jute fibers are cellulose, hemicellulose and lignin. Due to the differences in the nature of the amorphous hemicellulose and lignin and the semi-crystalline cellulose, a combination of characterization techniques is necessary to gain insight into the structural properties of jute fibers. While XRD is suitable for the characterization of the crystalline regions of cellulose fibers, FTIR spectroscopy can provide information about the chemical structures of both crystalline and amorphous components. Peaks in the diffraction patterns of jute samples (Figure 1) originate from the cellulose crystal planes. Cellulose is compiled of β-D-anhydroglucopyranose units (AGU) linked by β (1→4) ether bonds [14], forming chains, which are interconnected by hydrogen bonds. Arrangement of the hydrogen-bonded chains defines different polymorphs of cellulose.

Due to the high share of amorphous components, to accurately determine peak positions and intensities, d-spacings and crystallite sizes (D), the obtained diffractograms were deconvoluted (Figure 1 and Figure S1). Applying Equation (2) to the obtained XRD patterns (Figure 1), Z values were calculated (Table 2) for all three NWJ samples, proving that the predominant crystal polymorph of cellulose is Iβ, whose monoclinic unit cell is formed of two parallel cellulose chains interconnected by hydrogen bond network, with (1-10), (110) and (200) characteristic crystal planes (at 14.3–14.6°, at about 16.0°, and at 22.2–22.4°, respectively [13]). Using Segal’s equation (Equation (3)) crystalline indexes, indicators of the share of crystalline cellulose in the samples are calculated for all three jute samples (Table 2). Changes in the crystalline index values can be induced by two major processes: (a) removal of non-cellulose components, which leads to a reduction in the amorphous fraction in the sample, thereby increasing the relative share of the cellulose and consequently the crystalline index; (b) destruction of the hydrogen bond network of crystalline cellulose, leading to an increase in the amorphous cellulose share, and consequently lowering of the crystalline index value [15,16,17].

**Scheme 1:**
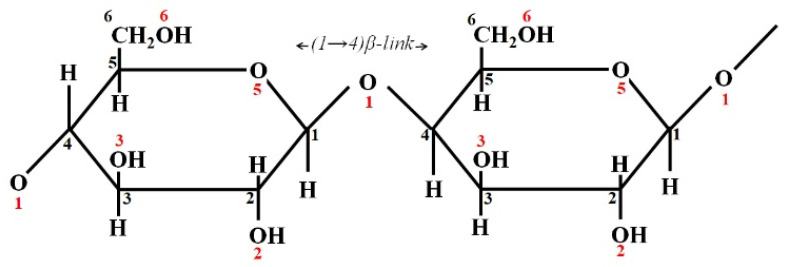
Molecular structure of a cellulose unit.

To understand the observed decreasing trend in Jw > Jo > Ja and the changes induced by alkali or oxidative treatment, defining the specific cellulose structure is of immense importance. A detailed analysis of the Iβ cellulose polymorph was first provided by Nishiyama et al. [18] and explained further by Eyley and Thielemans [14]. According to those authors, the surface of the nanocrystal is made up of cellulose chains directed along the (200) preferential lattice plane, where molecules are interconnected by hydrogen bonds between O3…O5 and O2…O6 hydroxyl groups. However, adjacent chains on the surface are linked by weak C–H…O hydrogen bonds and van der Waals interactions [14] placed along (110) and (1-10) planes, resulting in an arrangement such that hydroxymethyl and secondary hydroxyl groups (Scheme 1) are directed outside of the crystal [14]. These exposed groups are susceptible to reactions with H_2_O_2_ or NaOH, leading to the destruction of the hydrogen bond network, and depolymerization of the cellulose [19], which consequently leads to decreasing of the crystallite sizes along (110) and/or (1-10) planes (Table 2) and lowering of the crystallinity index.

The effect of the hydrogen peroxide is more pronounced along the (1-10) lattice plane, while the effect of NaOH is more expressed along the (110) lattice plane. An increase in the crystallite size along the (200) lattice plane in the NaOH-treated sample is indicative of cellulose chains reorientation along a longitudinal direction, an effect characteristic of alkali-treated cellulose material [20]. However, disruption of the hydrogen bond network induced by applied mild oxidative or alkali treatment did not result in a change in the cellulose polymorph, as no shift towards lower angles characteristic of the cellulose II polymorph (at 12.1° (1-10), 20.1° (110) and 21.9° (020) [17]) was detected.

**Figure 2:**
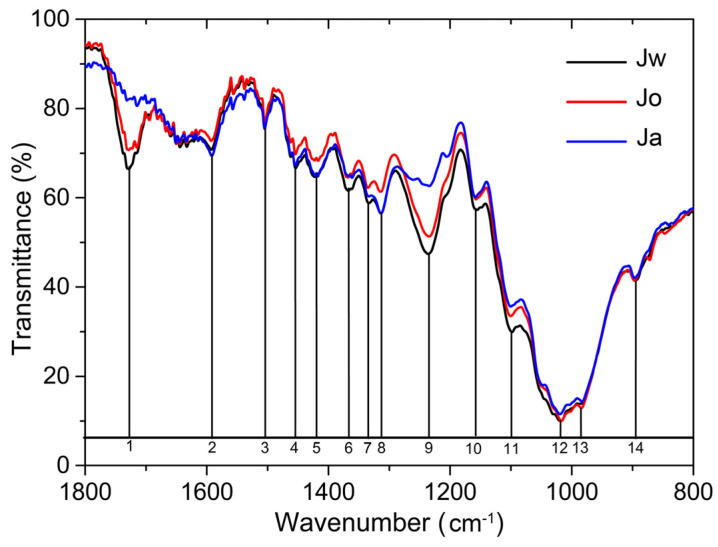
FTIR spectra of jute samples. Numbers 1–14 correspond to bands given in Table 3.

The influence of the chemical treatment on the amorphous components of the jute fibers is tracked by FTIR spectra (Figure 2 and Figure S2). To compare intensities of the bands after chemical treatments, spectra are normalized. Band assignments are done according to literature data [21] and are listed in Table 3.

Although the main expected effect of treatment with hydrogen peroxide was the removal of lignin through oxidative depolymerization and cleavage of C–C bonds [8,9,10], only slight decreases in the intensities of the bands related to aromatic skeletal vibration of lignin are observed, specifically bands centered at 1592 and 1506 cm^−^^1^. The effect of hydrogen peroxide treatment is visible on all bands originating from O–H banding vibrations of cellulose and hemicellulose (bands at 1315, 1335, 1420 and 1456 cm^−^^1^). This effect can be ascribed to the fact that H_2_O_2_ oxidizes secondary hydroxyl groups to ketone [22], which is in accordance with the results obtained from XRD findings; such a reaction would lead to the breaking of hydrogen bond networks in cellulose and lowering of the crystallinity index. Interestingly, hydroxymethyl groups of cellulose (C(6)…O(6)H)-related bands centered at 1020 and 985 cm^−^^1^ are not affected, although they could be oxidized to aldehyde or carboxylic acid upon reaction with hydrogen peroxide [22].

Effect of the NaOH treatment is, on the other hand, easily observable: two strong bands placed at 1727 cm^−^^1^, associated with C=O stretching vibration in the acetyl group of hemicellulose, and at 1236 cm^−^^1^, originating from C–O stretching vibration of carboxylic group of hemicellulose, have almost completely disappeared, confirming that alkali treatment of jute fibers even at low concentrations and for a relatively short times (3%, 30 min) leads to the dissolution of the amorphous hemicellulose. Again, bands related to hydroxymethyl groups of cellulose seem unaffected by alkali treatment.

**Figure 3:**
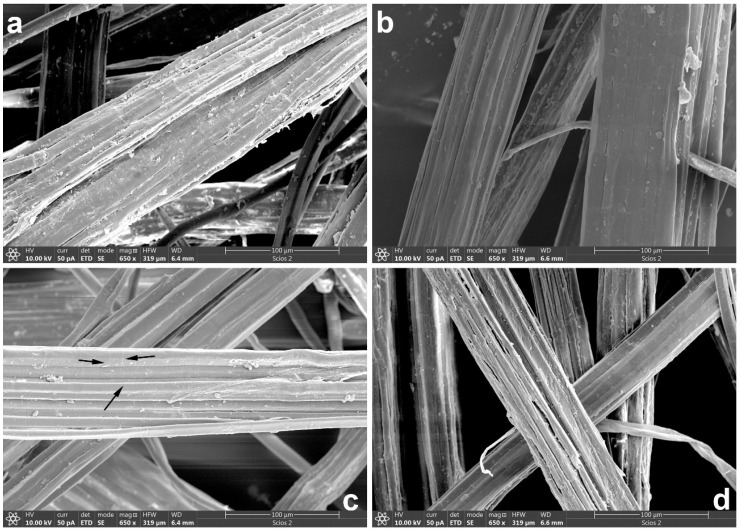
SEM images of jute samples: (**a**) untreated, (**b**) water-washed (Jw), (**c**) treated with hydrogen peroxide (Jo) and (**d**) alkali-treated (Ja). In panel (**c**), arrows indicate the formation of microcracks in the outer layer of the fibrils.

SEM micrographs (Figure 3) of treated samples are compared to evaluate the impact of the chemical treatment on the fiber surfaces. For the comparison, the SEM micrograph of the untreated, unwashed NWJ waste is also presented (Figure 3a). Untreated fabric is composed of fibers with a rough surface, covered with amorphous material and impurities. After simple washing with water (Figure 3b) most impurities and water-solubles are removed, resulting in fibers with relatively smooth surfaces, although amorphous components are still present. Similar cleaner surfaces of the fibers are characteristic for the jute samples treated with hydrogen peroxide (Figure 3c), but sporadically, the formation of microcracks in the outer layer of the fibrils (indicated by arrows) is noticeable, demonstrating that oxidative depolymerization of lignin has probably started to a minor extent. Finaly, alkali-treated jute fibers (Figure 3d) show the biggest change, as hemicellulose is moderately dissolved and removed (together with impurities and other minor components), resulting in partially or completely separated microfibrils.

### 3.2. NWJ Impregnated with nCN

**Figure 4:**
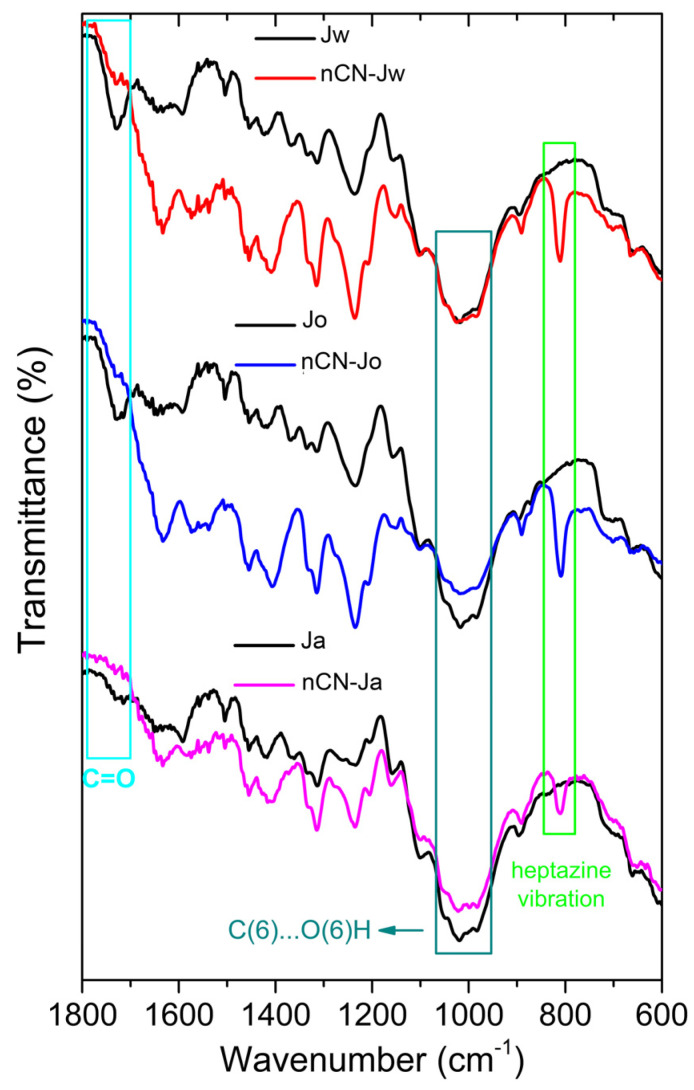
Comparative FTIR spectra of treated (black lines) and nCN-impregnated (colored lines) jute samples.

To clarify the mechanism of carbon nitride bonding to the jute surface, FTIR spectra of all three impregnated samples are compared with the spectra of unimpregnated counterparts (Figure 4 and Figure S3). Upon impregnation, the band at 1725 cm^−1^ (C=O stretching vibration in acetyl group and carboxylic group of hemicellulose) of nCN-Jw samples is completely suppressed, indicating that this group is the one of carbon nitride anchoring points to the Jw sample surface (Figure 4).

The same band is repressed in the FTIR spectrum of the nCN-Jo sample (Figure 4), but there is also significant reducing in the intensity of the bands related to (C(6)…O(6)H) vibrations of the hydroxymethyl groups of cellulose (centered at 1020 and 985 cm^−^^1^). The same band intensity reduction is characteristic of the FTIR spectrum of nCN-Ja sample where the overall intensity of the nCN-related bands is also much lower compared to the other two jute samples, quite expected if the acetyl and/or carboxylic groups of hemicellulose (partially removed upon alkali treatment in the Ja sample) are one of the points of carbon nitride bonding.

The entire region between 1700 and 1100 cm^−^^1^ is hidden by carbon nitride bands, preventing detailed analysis of the possible interaction between amino groups of carbon nitride and OH groups of jute samples. However, some conditional conclusions can be drawn: both oxidative and alkali treatments “neutralize” secondary hydroxyl groups of cellulose (first by oxidizing them to ketone [22], and later by ionization to alkoxide [19]), and the hydrogen bond network of the cellulose is disrupted in both chemically treated samples, Jo and Ja, leaving the hydroxymethyl group of the cellulose available for the interaction with the carbon nitride. These groups could be the main anchoring spots of the carbon nitride on the chemically treated jute surface. However, the same is not valid for the water-washed sample: impregnation has no effect on the hydroxymethyl-related band, probably because all secondary hydroxyl groups of cellulose are available for carbon nitride bonding, as well as hemicellulose.

**Figure 5:**
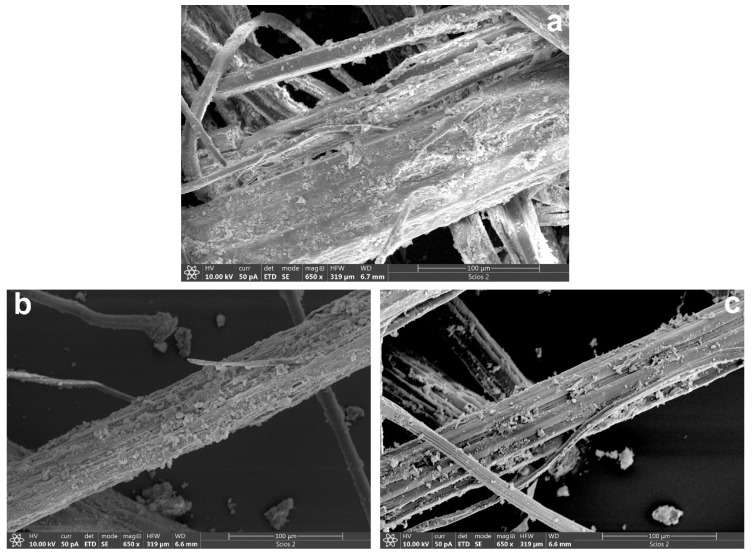
SEM images of impregnated jute samples: (**a**) nCN-Jw, (**b**) nCN-Jo and (**c**) nCN-Ja.

These differences in the surface chemistry of the jute fibers resulted in different carbon nitride distribution along the fiber upon impregnation (Figure 5).

SEM images (Figure 5a,b) of nCN-Jw and nCN-Jo samples underline the importance of the presence of hemicellulose, as carbon nitride is almost evenly distributed along the jute fibers, while for the alkali-treated sample nCN-Ja (Figure 5c), chunks of carbon nitride agglomerates are sporadically scattered on the jute fibers.

### 3.3. Photocatalytical Measurements

**Figure 6:**
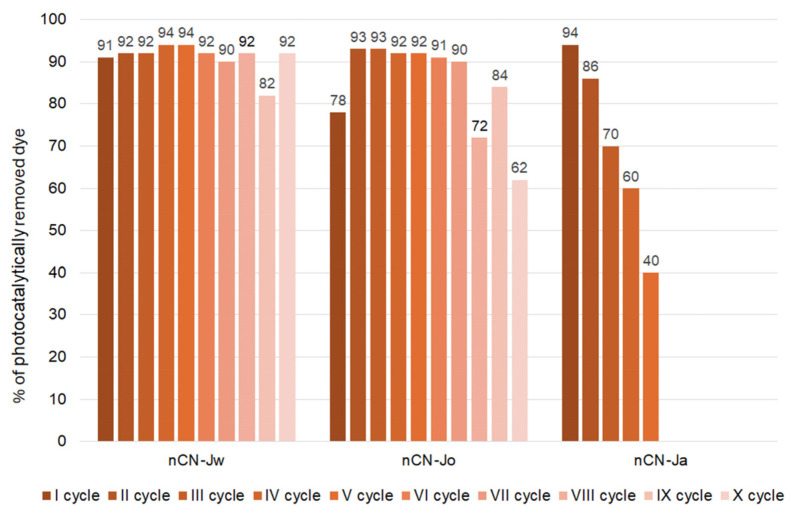
Percentage of photocatalytically removed dye using impregnated jute samples.

Finally, to evaluate the stability of carbon nitride-jute bonding and reusability of NWJ in multiple consecutive photocatalytic cycles, impregnated samples were probed in photocatalytic degradation of the textile dye AO7 under simulated solar light irradiation. Obtained results are presented in Figure 6, after 1 h of irradiation, without adsorption of the dye that can occur during the system standing in the dark for the initial adsorption/desorption equilibration step (cumulative results presented in Figure S4).

Photocatalytic degradation of AO7 on the nCN-Jw sample is constantly high through ten consecutive photocatalytic cycles, indicating the quality and stability of the carbon nitride–jute bonding, i.e., almost no carbon nitride is lost through repeated cycles, keeping efficacy of dye removal above 90%. The exception is the ninth cycle with 82%, a drop that can be explained by cumulative contamination of the carbon nitride surface with the intermediates of the photocatalytic reaction.

Similar behavior is expressed by the nCN-Jo sample. After the first sluggish cycle, in the next six cycles, the percentage of the removed dye is above 90%. However, the last three cycles experienced a sharp decline in effectiveness reaching just 62% in the tenth cycle. Finally, after outstanding 94% of the dye removed in the first cycle, in the next four cycles, the effectiveness of the nCN-Ja sample suffered a constant sharp decline, reaching only 40% in the final fifth cycle. Keeping in mind the poor distribution of the carbon nitride on the alkali-treated fiber (Figure 5c), this result is not surprising. However, another point regarding the instability of carbon nitride–jute bonding in chemically treated samples also must be considered: nCN is not a selective photocatalyst, meaning that photogenerated charges formed upon its illumination form radicals (by a mechanism explained in detail in [11]) which in turn can react non-selectively with all present species, not just AO7, including the carbon nitride–jute bond. The decline in the photocatalytic effectiveness of nCN-Jo and nCN-Ja samples can be a consequence of the breakage of the of the bond formed between amino group of the carbon nitride and hydroxymethyl group of the cellulose, characteristic for both samples as confirmed by FTIR spectra. Such a hypothesis is further supported by the constantly high photocatalytic effectiveness of the nCN-Jw sample, for which it is expected that carbon nitride is bonded to the fiber’s surface through hydrogen bonds with secondary OH groups of cellulose and hemicellulose.

**Figure 7:**
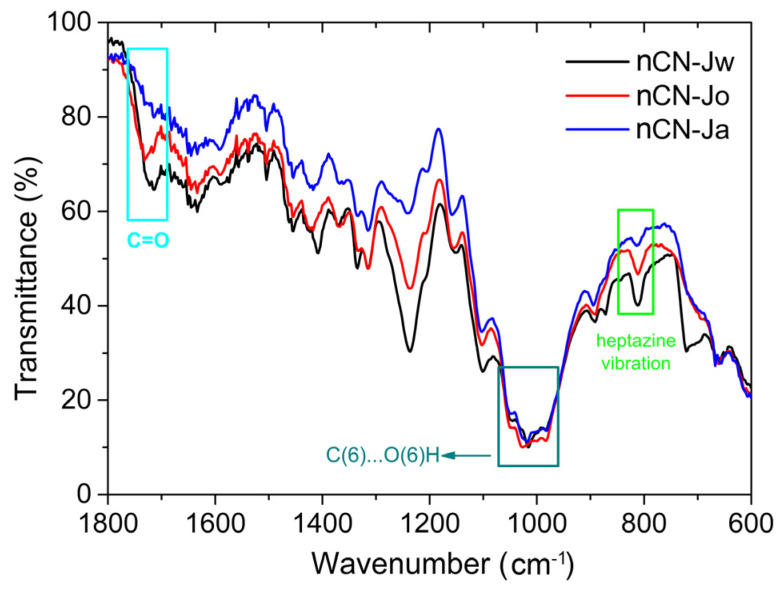
FTIR spectra of nCN-J samples after ten (nCN-Jw and nCN-Jo) or five (nCN-Ja) photocatalytic cycles.

Additionally, FTIR spectra of the impregnated samples recorded after the final photocatalytic experiments (Figure 7 and Figure S5) revealed that:

- The band at ~1725 cm^−^^1^ is recovered in the nCN-Jw and nCN-Jo samples, pointing to the breakage of the carbon nitride–hemicellulose bond.
- Intensities of the bands related to hydroxymethyl group vibrations (at 1020 and 985 cm^−^^1^) are fully recovered in the FTIR spectra of the nCN-Jo and nCN-Ja samples, indicating the detachment of carbon nitride from the cellulose.
- In all three spectra, the band at ~ 815 cm^−^^1^ (characteristic vibration of heptazine) is present, proving the presence of the carbon nitride in the impregnated samples even after multiple photocatalytic cycles, but with significant differences in the intensities, which can be correlated to the lowering of the photocatalytic effectiveness and stability in the order Jw > Jo > Ja.

## 4. Conclusions

Post-industrial jute waste was pre-treated with hydrogen peroxide or NaOH and then compared with water-washed samples. Results from the XRD analysis revealed the profound effect that chemical treatment had on the cellulose fibers, as both chemical treatments led to the destruction of the hydrogen bond network between cellulose chains: oxidative treatment was mainly expressed along the (1-10) lattice plane, while alkali treatment was more pronounced along the (110) lattice plane of the cellulose. Therefore, the mechanism of the carbon nitride bonding to the jute fiber surface changed significantly: while in the water-washed sample, the major presumed anchoring points are secondary hydroxyl groups of the cellulose, in the chemically treated samples, these are hydroxymethyl groups of cellulose, as confirmed by FTIR spectra. SEM images revealed the differences in the fiber surface chemistry and the bonding mechanism; carbon nitride showed the worst distribution over NaOH-treated samples. Photocatalytic efficiency and stability of the impregnated samples declined in the order Jw > Jo > Ja.

Results obtained in this study can serve as a guideline for studies related to the usage of any cellulose-based material as support for a photocatalyst and underline the importance of a thorough understanding of the chemical and structural processes involved upon chemical treatment of textile fibers.

Finally, we have demonstrated that jute waste solely subjected to water-washing to eliminate impurities and water-soluble compounds, exhibits notable potential as a support for nCN photocatalysts. The nCN-Jw sample showed continuously high degradation rates over ten cycles, with minimal loss of carbon nitride and a dye removal percentage consistently above 90%.

## Figures and Tables

**Table 1:** Moisture content and weight loss of NWJ samples.

Sample	Moisture Content (%)	Weight Loss (%)
Jw	7.6 ± 0.3	1.3 ± 0.1
Jo	7.6 ± 0.3	3.5 ± 0.4
Ja	7.6 ± 0.2	5.1 ± 0.4

**Table 2:** Parameters calculated from deconvoluted XRD patterns.

Sample	Z	Cr.I. (%)	d(1-10) (nm)	d(110) (nm)	d(200) (nm)	D(1-10) (nm)	D(110) (nm)	D(200) (nm)
Jw	−11	75.0	0.60	0.53	0.40	4.14	2.58	2.59
Jo	−11	72.9	0.60	0.53	0.40	3.86	2.64	2.56
Ja	−11	70.6	0.60	0.53	0.40	3.91	2.16	2.73

**Table 3:** FTIR spectra band assignment.

	Wave Number (cm^−1^)	Band Assignment
1	1727	HC, C=O stretching in acetyl group and carboxylic group
2	1592	L, Aromatic skeletal vibration HC, (COO^−^ stretching)
3	1506	L, Aromatic skeletal vibration
4	1456	Ce, HC, O–H in-plane bending
5	1420	Ce, HC, O–H in-plane bending L, C–H bending in CH_3_
6	1365	Ce, HC, C–H bending
7	1335	Ce, HC, O–H in-plane bending
8	1315	Ce, HC, O–H in-plane bending
9	1236	HC, C–O stretching in carboxylic acid
10	1155	Ce, HC, C–O–C antisymmetric stretching
11	1100	Ce, HC, C(2)…O(2)H stretching
12	1020	Ce, C(6)…O(6)H stretching
13	985	Ce, C(6)…O(6)H stretching
14	896	Ce, HC, antisymmetric vibration at the β-glycosidic linkage

Abbreviations: HC—hemicellulose, L—lignin, Ce—cellulose.

## Data Availability

The original contributions presented in the study are included in the article/Supplementary Material; further inquiries can be directed to the corresponding authors.

## Supplementary Materials

The following supporting information can be downloaded at: https://www.mdpi.com/article/10.3390/polym16141989/s1, Table S1: Properties of acid orange 7; Figure S1: XRD patterns of the NWJ samples: Jw (water-washed), Jo (treated with hydrogen peroxide) and Ja (alkali-treated); Figure S2: FTIR spectra of the NWJ samples; Figure S3: Comparative FTIR spectra of treated (black lines) and nCN impregnated (colored lines) jute samples; Figure S4: Percentage of the removed dye using nCN-J samples; Figure S5: FTIR spectra of nCN-J samples after ten (nCN-Jw and nCN-Jo) or five (nCN-Ja) photocatalytic cycles. Ref. [23] is listed in References of main text.
