# Supplementary material for: Postindustrial Jute Waste as a Support for Nano-Carbon Nitride Photocatalyst: Influence of Chemical Pretreatment
Source: Polymers (Basel). 2024 Jul 11;16(14):1989. doi: 10.3390/polym16141989 (PMC11280916; doi:10.3390/polym16141989)
Supplement: Supplementary file 1 [file polymers-16-01989-s001.zip › polymers-3082596-supplementary.pdf]

# Supplementary Materials

## Postindustrial Jute Waste as a Support for Nano-Carbon Nitride Photocatalyst: Influence of Chemical Pretreatment

Milica V. Carević[1*], Tatjana D. Vulić[1], Zoran V. Šaponjić[2], Nadica D. Abazović[1*], Mirjana I. Čomor[1]

[1]Vinča Institute of Nuclear Sciences, National Institute of the Republic of Serbia, University of Belgrade, Mike Petrovića Alasa 12-14, 11351, Vinča, Belgrade, Serbia; milicab@vin.bg.ac.rs (M.V.C.); tanja030@vin.bg.ac.rs (T.D.V.); kiki@vin.bg.ac.rs (N.D.A.); mirjanac@vin.bg.ac.rs (M.I.Č.);

[2]Institute of General and Physical Chemistry, Belgrade, Studentski Trg 12/V, 11158, Belgrade, Serbia; zsaponjic@iofh.bg.ac.rs (Z.V.Š);

[*]milicab@vin.bg.ac.rs (M.V.C.); kiki@vin.bg.ac.rs (N.D.A.)

## Experimental section

### *nCN synthesis*

5 g of urea were calcined at 550 °C for 2 hours with a heating rate of 5 °C/min in a semi-covered ceramic crucible. Once the resulting powder had cooled to room temperature, 250 mg of it was placed in an open crucible and heated to 500 °C at a rate of 5 °C per minute. It was then maintained at 500 °C for two hours. The synthesized sample was ground and collected for subsequent use.

### *Photocatalytic activity test*

In a typical test, nCN-J sample was immersed in 100 mL of AO7 aqueous solution ($C_0$=10 ppm). The experiments were performed under continuous magnetic stirring and bubbling with $O_2$, at natural pH, room temperature and atmospheric pressure. Following an hour of stirring the mixture in the dark, Osram vitalux lamp which simulates solar irradiation (300 W, white light: UVB radiated power from 280 to 315 nm 3.0 W; UVA radiated power 315–400 nm 13.6 W; the rest is visible light and IR) was turned on. The distance between the reaction mixture and the light source

was set at 30 cm. Aliquots (2 mL) were regularly collected, centrifuged (12000 rpm, 30 min) and supernatants were analysed by UV-Vis spectroscopy. Finally, the $C/C_0$ vs. time was plotted (C is the concentration of the dye solution after a certain time of illumination and $C_0$ is the initial concentration of the dye solution determined for a wavelength equal to 485 nm). The photolysis and absorption studies were conducted under the same conditions as photocatalytic experiments, but without a photocatalyst (photolysis) or without irradiation (absorption).

Table S1. Properties of the **Acid Orange 7**

| | |
|---|---|
| **Structure** | 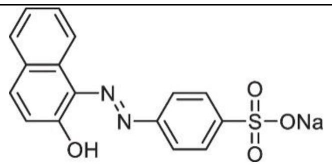 |
| **type** | azo-dye |
| **$\lambda_{max}$ (nm)** | 485 |
| **$pK_a$** | ~8.26 |
| **Applications** | Silk, wool, paper, leather dyeing. |

## Results and Discussion

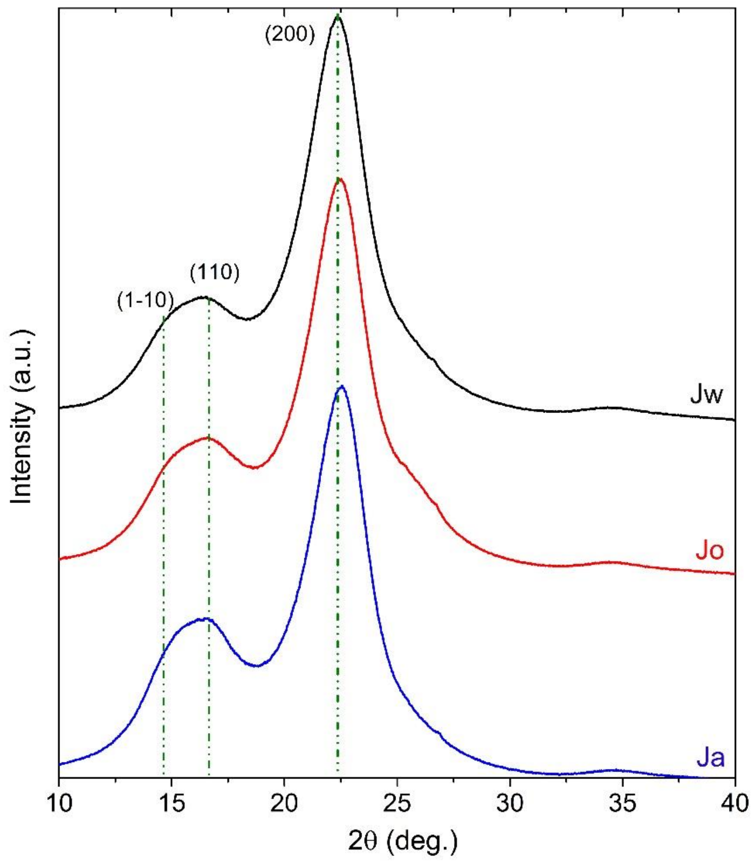


**Figure S1.** XRD patterns of the NWJ samples: Jw (water-washed), Jo (treated with hydrogen-peroxide) and Ja (alkali-treated).

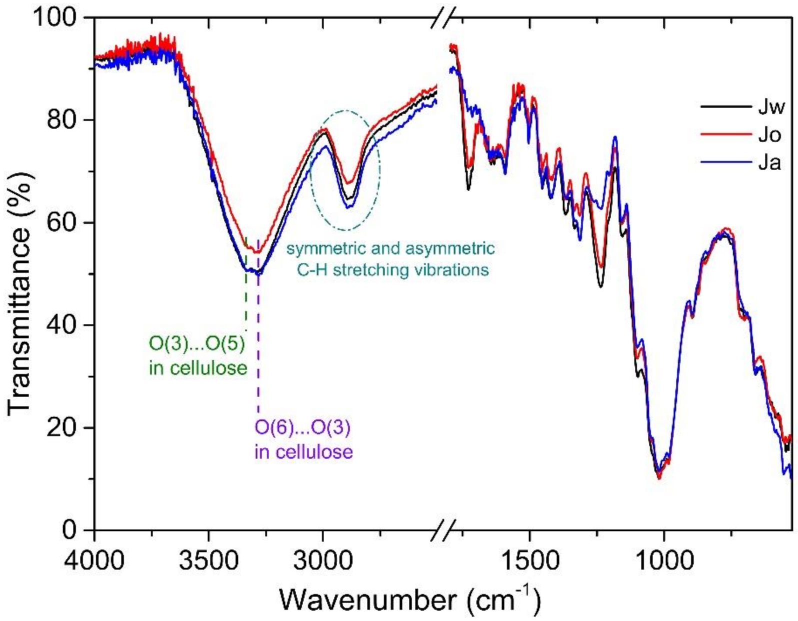


**Figure S2.** FTIR spectra of the NWJ samples.

Wide band stretching from ~3700 $cm^{-1}$ to ~ 3000 $cm^{-1}$ is characteristic for all studied samples. It is a superposition of the vibrations originating from the intramolecular hydrogen bonding O(3)H…O(5) (3375-3340 $cm^{-1}$) and intermolecular hydrogen bonding O(6)H…O(3) (3310-3230 $cm^{-1}$) in cellulose. [23] Band placed from ~3000 $cm^{-1}$ to ~2700 $cm^{-1}$ originates from the symmetric

and asymmetric C-H stretching vibrations of the methyl and methylene groups.

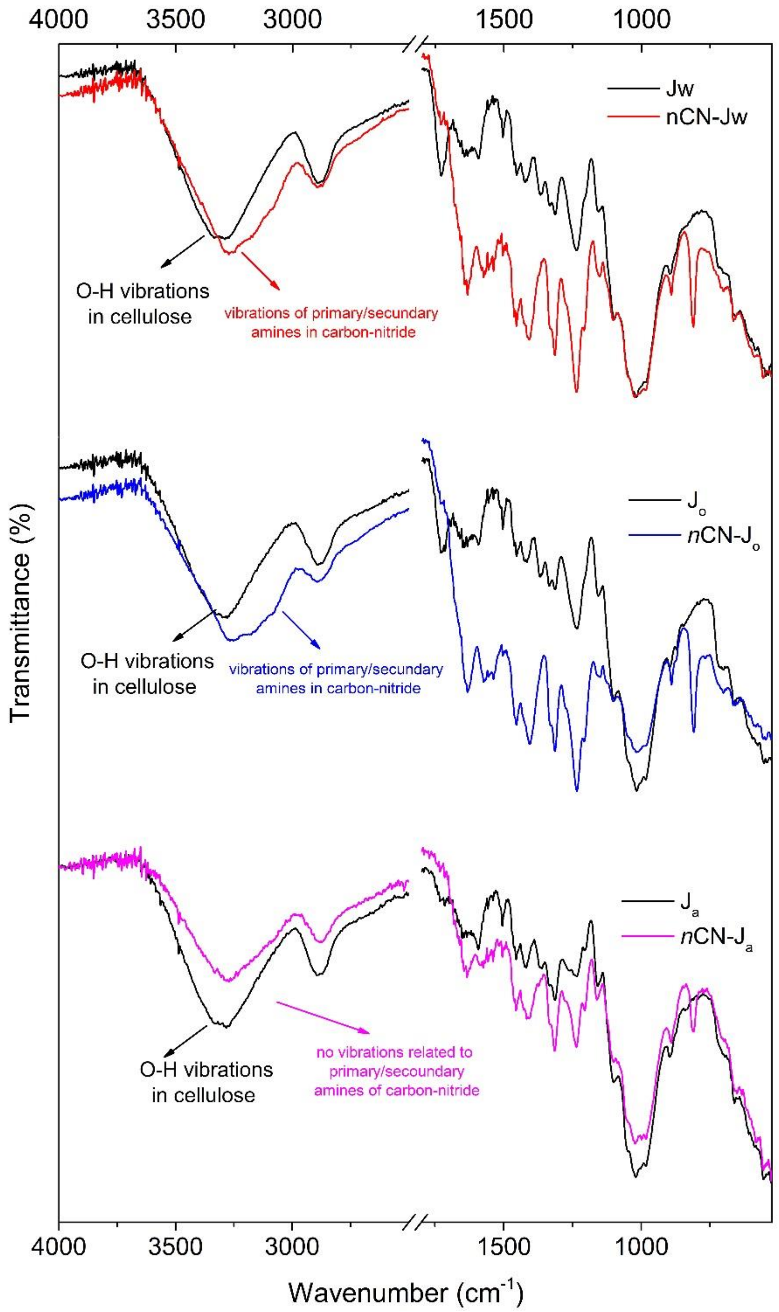


**Figure S3.** Comparative FTIR spectra of treated (black line) and *n*CN impregnated (coloured line) jute samples.

In the FTIR spectra of the impregnated nCN-Jw and nCN-Jo samples new wide band emerges with the maximum at ~ 3290 $cm^{-1}$, partially overlapping with the band originating from the O-H intramolecular and intermolecular vibrations of the cellulose. The new band originates from the N-H vibrations of the amino and imino groups of the carbon nitride. However, it is completely

absent in the FTIR spectrum of the nCN-Ja sample, underlining poor bonding of the nCN on the surface of the alkali treated jute.

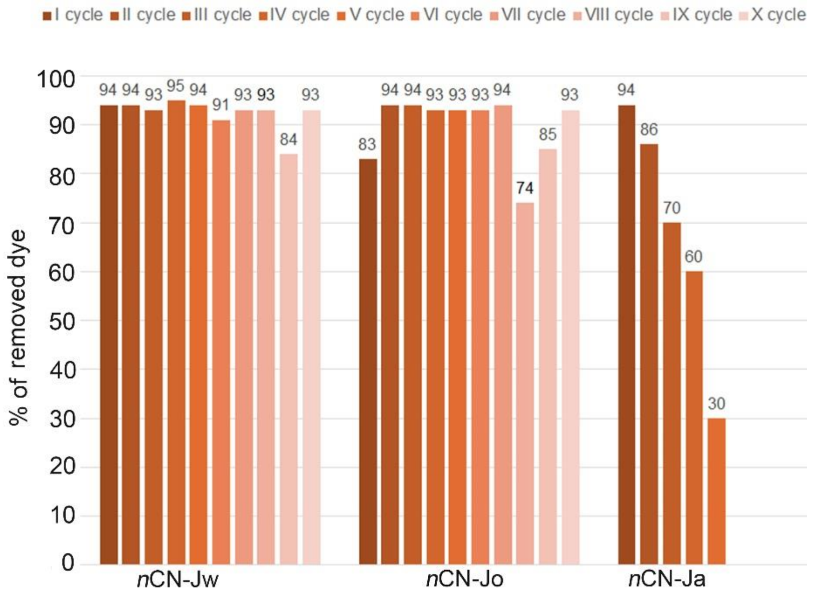


**Figure S4.** Percentage of the removed dye using nCN-J samples.

Prior to the photocatalytic experiments, impregnated samples immersed in the 100 mL of the 10 ppm solution of AO7, were kept 1h in the dark in order to achieve adsorption/desorption equilibrium. In Figure S4 cumulative results (adsorption and photocatalytic degradation) of the dye removal in the multiple consecutive cycles are presented.

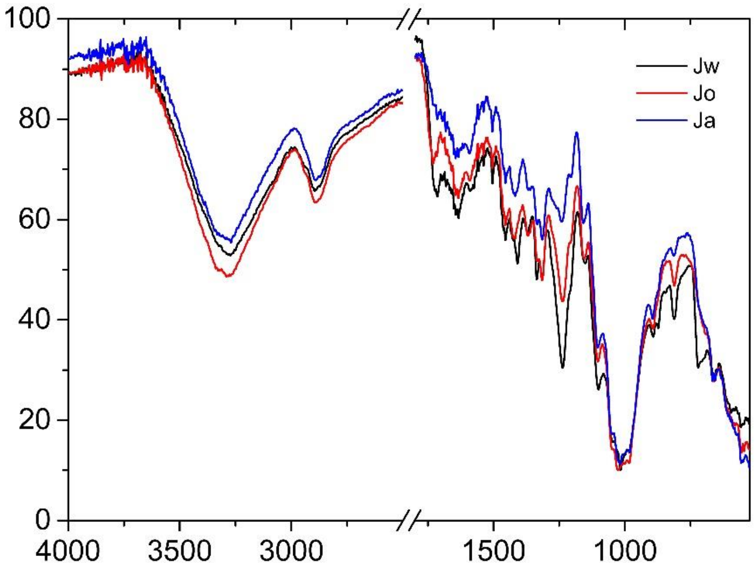


**Figure S5.** FTIR spectra of nCN-J samples after ten (nCN-Jw and nCN-Jo) or five (nCN-Ja) photocatalytic cycles

References:

[23] Sang Youn Oh, Dong Il Yoo, Younsook Shin and Gon Seo, FTIR analysis of cellulose treated with sodium hydroxide and carbon dioxide, *Carbohydrate Research*, 340 (2005) 417–428.
